# Butyrate ameliorates DSS-induced ulcerative colitis in mice by facilitating autophagy in intestinal epithelial cells and modulating the gut microbiota through blocking the PI3K-AKT-mTOR pathway

**DOI:** 10.1371/journal.pone.0337214

**Published:** 2025-12-11

**Authors:** Ruifang Li, Yan Sun, Drolma Kelsang, Mingzhi Feng, Hanhui Cai, Jun Zhang

**Affiliations:** 1 Center for General Practice Medicine, Department of Gastroenterology, Zhejiang Provincial People’s Hospital, (Affiliated People’s Hospital, Hangzhou Medical College), Hangzhou, Zhejiang, P.R China; 2 Emergency and Critical Care Center, Intensive Care Unit, Zhejiang Provincial People’s Hospital, (Affiliated People’s Hospital, Hangzhou Medical College), Hangzhou, Zhejiang, P.R China; 3 Department of Gastroenterology, The First Affiliated Hospital of Zhejiang Chinese Medical University (Zhejiang Provincial Hospital of Traditional Chinese Medicine), Hangzhou, Zhejiang, P.R China; University of Illinois at Chicago, UNITED STATES OF AMERICA

## Abstract

**Background:**

Ulcerative colitis (UC) is an idiopathic, chronic inflammatory disease of the colon that imposes a significant global public health burden due to its recurrent and refractory nature. The therapeutic effects of butyrate on UC have been documented previously, but its specific mechanisms remain unclear.

**Objective:**

This study aimed to investigate the effects and potential mechanisms of butyrate in dextran sulfate sodium (DSS)-induced UC.

**Methods:**

A UC mouse model was induced utilizing 3% DSS, followed by interventions of 300 mg/kg, 600 mg/kg, or 1200 mg/kg of butyrate. The fecal condition and colonic pathology of the mice were observed, along with measurements of body weight and colon length. The expression of serum inflammatory factors, autophagy-related proteins, and the PI3K/AKT/mTOR pathway-related proteins were detected. Additionally, the impact of butyrate on the mouse gut microbial communities was evaluated using 16S rRNA sequencing technology.

**Results:**

It was demonstrated that butyrate effectively alleviated UC symptoms, including bloody stools, weight loss, and colon shortening. Furthermore, butyrate reduced colonic tissue damage, decreased the expression of TNF-α, IL-1β, and IL-6, and significantly upregulated Beclin-1, Atg5, and the LC3II/I ratio, while reducing P62 expression. Butyrate also decreased the relative expression of p-PI3K/PI3K, p-Akt/Akt, and p-mTOR/mTOR. 16S rRNA sequencing results revealed that butyrate improved the intestinal microbial community structure by inhibiting harmful bacteria and accelerating the growth of beneficial bacteria.

**Conclusion:**

This study suggests that butyrate may alleviate DSS-induced UC in mice by blocking the PI3K/AKT/mTOR pathway to facilitating autophagy in intestinal epithelial cells and by modulating the gut microbiota.

## Introduction

Ulcerative colitis (UC) is a chronic non-specific inflammatory disorder predominantly affecting the colon and rectum, with lesions generally confined to the mucosa and submucosa of the colorectum [1]. Studies indicate a steady increase in the incidence and prevalence of ulcerative colitis (UC) over the past two decades [2]. UC is characterized by a protracted and relapsing course [3], a feature that significantly impacts patients’ quality of life and long-term prognosis [4]. Current clinical management relies primarily on 5-aminosalicylic acid (5-ASA) drugs, steroids, immunosuppressants, and biologics, which frequently demonstrate suboptimal efficacy and may induce adverse effects—including renal impairment, hypersensitivity reactions, and immunosuppressive effects—particularly with long-term use [5,6]. Consequently, the search for natural substances with minimal side effects for preventing and treating UC remains a central focal of contemporary research.

Mounting evidence indicates that gut microbiota dysbiosis is pivotal in the pathogenesis of UC [7,8]. Although fecal microbiota transplantation (FMT) has shown promising efficacy in treating UC by modifying the gut microbial composition of patients in a number of clinical studies [9], the precise mechanisms underlying this effect remain poorly understood. Recent studies also indicate that impairments in the autophagic processes of intestinal epithelial cells can influence both the structure and functionality of the gut microbiota, thereby influencing the immune regulation and inflammatory responses within the intestine [10–13]. Findings from genetic studies highlight that defects in autophagy-related genes in these cells are linked to decreased microbial diversity and expansion inflammatory and potentially pathogenic bacterial populations [14]. Furthermore, the PI3K-AKT-mTOR pathway is known to modulate autophagy in these cells [15], its precise role in driving microbial dysbiosis in UC patients remains unclear and warrants further exploration.

Butyrate (BA), a short-chain fatty acid generated by the fermentation of dietary fibers by intestinal microbes, is vital for gut health [16–18]. Compared to healthy individuals, UC patients have a significantly reduced butyrate-producing microbial population in the gut, with lower levels of butyrate in the feces [19]. Supplementing BA may positively affect the modulation of the gut microbiota in UC patients [20], though this is somewhat controversial [21]. It is imperative to delve deeper into how BA influences the gut microbiota to better understand its potential therapeutic role in UC.

In conclusion, an established link exists between BA, autophagy in intestinal epithelial cells (IECs), and gut microbiota dysbiosis. However, the intricate interplay between BA, IEC autophagy, and microbial composition—along with the specific protective mechanisms involved—remain unclear. We therefore hypothesized that BA induces promotes IEC autophagy and reshapes the intestinal microbiota, thereby attenuating intestinal inflammation in UC. To test this hypothesis, we employed a dextran sulfate sodium (DSS)-induced murine UC model. We assessed the protective effects of BA by administering varying BA doses and evaluating changes in body weight, colon length, and inflammatory cytokine level. Furthermore, we analyzed gut microbiota composition and structure via 16S rRNA sequencing and examined its correlation with autophagy markers. Key proteins in the autophagy pathway and intestinal protein expression were quantified to elucidate the molecular mechanisms of BA action. This study elucidates the protective effects and underlying mechanisms of BA against DSS-induced UC, providing a theoretical and experimental foundation for novel UC therapeutic strategies.

## Materials and methods

### Experimental reagents

DSS was purchased from MP Biomedicals (0216011080, California, USA). BA was obtained from Sigma-Aldrich (B5887, Missouri, USA). 5-ASA was sourced from Aladdin (A129982, Shanghai, China). Antibodies for AKT, phospho-AKT (p-AKT), LC3II/I, and Atg5 were acquired from Cell Signaling Technology (#4691, #4060, #12741, and #12994, Boston, USA). mTOR antibody was purchased from Proteintech (81670–1-RR, Chicago, USA), and phospho-mTOR (p-mTOR) from Hu’an (HA600094, Hangzhou, China). PI3K, P62, and Beclin-1 antibodies were obtained from ABclonal (A19742, A19700, and A21191, Wuhan, China), and phospho-PI3K (p-PI3K) from Bioss (bs-5570R, Beijing, China). Mouse tumor necrosis factor-α (TNF-α), mouse interleukin-1β (IL-1β), and mouse interleukin-6 (IL-6) ELISA kits were procured from Wuhan Elabscience Biotechnology Co., Ltd. (E-EL-M3063, E-EL-M0037c, and E-EL-M0044c, Wuhan, China).

### Experimental animals

Thirty-six 5-week-old male SPF-grade C57BL/6 mice (weight: 20 ± 2 g) were acquired from Hangzhou Medical College (Production license: SCXK (Zhe) 2019–0002, Hangzhou, China). These mice were accommodated at the Animal Experiment Center of Hangzhou Medical College (Use license: SYXK (Zhe) 2019–0011). Environmental conditions were meticulously controlled, maintaining a relative humidity of 45–55%, a temperature of 24 °C (± 2 °C), and a 12-hour light/dark cycle. The mice had free access to water and standard lab feed throughout the study. The experimental protocols involving these animals were reviewed and approved by the Zhejiang Province Experimental Animal Center’s Animal Ethics Committee (Approval number: ZJCLA-IACUC-20010192).

### Animal grouping and model establishment

**Fig 1:**
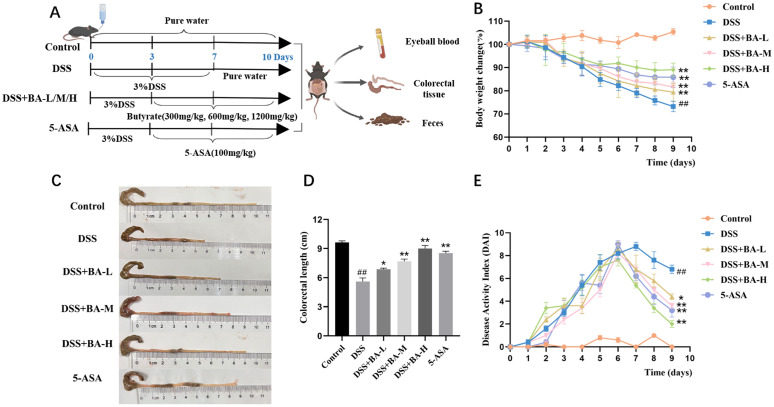
BA protects against DSS-induced colitis in mice. **(A)** Experimental timeline. **(B)** Body weight changes. **(C)** Colon length. **(D–E)** Disease activity index (DAI). Data are Mean ± SEM, n = 6; one-way ANOVA. ##P < 0.01 vs. Control; *P < 0.05, **P < 0.01 vs. DSS group. Groups: Control, DSS (DSS-induced model), DSS-BA-L (butyrate low-dose), DSS-BA-M (butyrate medium-dose), DSS-BA-H (butyrate high-dose), 5-ASA (5-aminosalicylic acid).

Thirty-six C57BL/6 mice were randomized into six groups: control group (Control), model group (DSS), low-dose butyrate group (DSS + BA-L, 300 mg/kg), medium-dose butyrate group (DSS + BA-M, 600 mg/kg), high-dose butyrate group (DSS + BA-H, 1200 mg/kg), and a positive control group treated with 5-ASA (100 mg/kg). All groups except the control group were administered 3% DSS in drinking water for seven days to establish the UC model. From day 3 of model establishment, the DSS BA-L, DSS BA-M, DSS BA-H, and 5-ASA groups were treated via oral gavage with the corresponding drugs for 7 consecutive days. During the experiment, daily records of body weight and changes in fecal characteristics were maintained. The severity of UC in mice was assessed utilizing a Disease Activity Index (DAI) scoring chart (Table 1). On the 11th day of the experiment, mice were anesthetized with sodium pentobarbital (50 mg/kg), followed by blood sample collection. Subsequently, the mice were euthanized to harvest the colorectal tissues and fecal samples (Fig 1A).

**Table 1:** Disease activity index score.

Score	Weight loss (%)	Stool characteristics	Hematochezia
0	None	Normal	Negative
1	1-5	Soft but still formed	1+
2	5-10	Loose	2+
3	10-15	Loose stools	3+
4	≥15	Watery stool	Naked eye bloody stool

### Histological examination

The colon tissue was fixed with 4% paraformaldehyde, embedded and sectioned using paraffin. The sections (5 µm) were then deparaffinized with xylene and stained with hematoxylin and eosin stain (H&E). Changes in colonic tissue structure, glands and cup cells were assessed using microscopic observation. The degree of tissue damage was assessed using the Histological Injury Score (HI) (Table 2).

**Table 2:** Histological injury scoring criteria.

Score	Inflammation	Depth of lesion	Crypt deatruction
0	Not have	Not have	Not have
1	Mild	Mucosal muscle layer	Base 1/3
2	Moderate	Muscular layer	Base 2/3
3	Severe	Serosal layer	Only complete surface epithelium
4	——	——	Complete epithelial destruction

### ELISA for serum inflammatory markers

Mouse blood samples collected from the orbits were allowed to stand at 4°C for 2–3 hours before being centrifuged at 12,000 rpm for 15 minutes to obtain the supernatant. The levels of inflammatory markers TNF-α, IL-1β, and IL-6 in the serum were quantitatively assessed employing ELISA kits.

### 16S rRNA gene sequencing

Genomic DNA was isolated from fecal samples of mice, and its structural integrity was verified through 1% agarose gel electrophoresis. Both the concentration and purity of the isolated DNA were quantified before using it as a template for the PCR amplification of the V3-V4 regions of the 16S rRNA gene, which were identified by barcode-tagged primers. Subsequently, the PCR products were retrieved, purified, and utilized to prepare a sequencing library. This library was then sequenced on an Illumina Miseq PE300 system. Quality control of the raw paired-end reads was conducted using fastp software, and the reads were merged using FLASH software. Operational taxonomic units (OTUs) were clustered with a 97% similarity threshold using UPARSE software, and chimeric sequences were excised. Taxonomic classification of the OTUs was carried out against the Silva 16S rRNA gene database version 138, applying an RDP classifier with a 70% confidence level. The composition and variations of the microbial communities at different taxonomic levels were examined utilizing the cloud-based bioinformatics platform provided by Majorbio, Shanghai.

### Western blot

Approximately 20 mg of colon tissue was placed in a 2 mL centrifuge tube containing RIPA lysis buffer, protease inhibitors, phosphatase inhibitors, and grinding beads. The tissue was homogenized using a tissue homogenizer and then incubated on ice for 30 minutes before being centrifuged at 12,000 rpm for 15 minutes to separate the supernatant. Protein levels in the supernatant were quantified utilizing a BCA Protein Assay Kit. The proteins were then resolved through SDS-PAGE and electrotransferred onto a PVDF membrane via a wet transfer technique. This membrane was blocked with non-fat milk at ambient temperature for an hour and then incubated overnight at 4°C with primary antibodies targeting PI3K, p-PI3K, AKT, p-AKT, mTOR, p-mTOR, Beclin-1, P62, LC3II/I, and Atg5. Following this, the membrane was incubated with horseradish peroxidase-labeled secondary antibodies at ambient temperature on a shaker for an hour. Visualization of the protein bands was achieved using ECL reagent, and Image J software was employed for analysis.

### Immunofluorescence staining

Paraffin-embedded mouse colon tissue sections were first deparaffinized and rehydrated, followed by antigen retrieval using EDTA buffer in a microwave. The sections were then blocked with 10% goat serum at ambient temperature for 30 minutes. Overnight incubation at 4°C was carried out with primary antibodies specific for LC3, Beclin-1, and P62. Subsequent to TBST washing, the sections were treated with secondary antibodies for an hour at ambient temperature and then stained with DAPI to visualize nuclei. The prepared slides were analyzed under a fluorescence microscope to capture detailed images.

### Transmission electron microscopy (TEM)

A 1 mm^3^ piece of colon tissue was fixed with electron microscopy fixative and rinsed with phosphate buffer. The tissue was then fixed again at room temperature for 2 hours in 1% osmium tetroxide in the dark. Following room temperature dehydration, the tissue was embedded in Epon 812 resin in a 37°C oven overnight. The following day, the resin block was further polymerized at 60°C for 48 hours. Ultra-thin sections were then precisely cut with an ultramicrotome, collected on 200-mesh copper grids, and sequentially stained—first with 2% uranyl acetate in ethanol for 15 minutes in darkness, followed by lead citrate under carbon dioxide-free conditions for 10 minutes. Once stained, the sections were left to dry overnight at ambient temperature in a grid box. The prepared sections were finally examined and imaged employing a transmission electron microscope.

### Statistical analysis

Data analysis and plotting of statistical graphs were done using GraphPad Prism 9.5 and data were expressed as mean ± standard error (Mean ± SEM). Comparisons between groups were analyzed using one-way ANOVA and the least significant difference (LSD) test. Spearman correlation analysis was used to assess the correlation between intestinal flora and autophagy. p < 0.05 indicated statistical significance.

## Results

### Protective effects of BA on DSS-induced UC in mice

In the control group, the mice had smooth and shiny fur, normal food intake and feces. After three days of continuous intake of 3% DSS, the mice showed a gradual roughening of fur, decreased vigor, reduced feeding, and thin feces with occult blood. On the fourth day, some of the mice had worsened symptoms and showed obvious bloody stools. Compared with the control group, mice in the DSS group showed weight loss (Fig 1B), significantly shorter colon length (9.63 ± 0.16 cm vs 5.60 ± 0.37 cm), and colorectal bleeding. Compared with the mice in the DSS group, the shortening of the colon in the mice treated with BA intervention and 5-ASA was significantly improved (Fig 1C and D), and the mice showed a trend of recovery in body weight (Fig 1B), with the most significant effect in the DSS + BA-H group. In addition, the DAI scores of mice in the DSS group (4.9 ± 1.07) were significantly higher than those in the DSS + BA-L group (4.24 ± 0.89), DSS + BA-M group (3.58 ± 0.86), DSS + BA-H group (3.80 ± 0.86), and 5-ASA group (3.76 ± 0.94) (Fig 1E).

### BA alleviates DSS-induced colonic inflammation in UC mice

**Fig 2:**
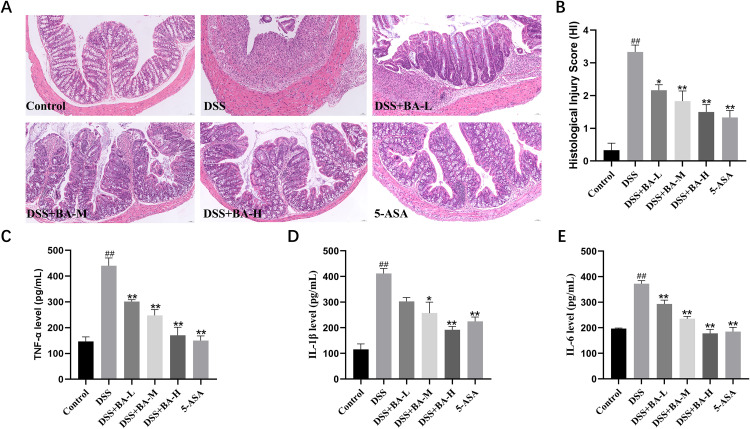
BA ameliorates colitis in DSS-induced mice. **(A)** Colon histology (H&E staining, 100×). **(B)** Histopathological score. **(C–E)** ELISA of TNF-α **(C)**, IL-1β **(D)**, and IL-6 **(E)** in colon tissue. Data are Mean ± SEM, n = 6; one-way ANOVA. #P < 0.05, ##P < 0.01 vs. Control; *P < 0.05, **P < 0.01 vs. DSS group.

Histological examination revealed that in the control group, the mucosal layer of the mouse colon was intact, with well-organized and regular glands, and no signs of submucosal congestion or edema were observed. In contrast, the DSS group exhibited disrupted mucosa and glands, marked submucosal congestion and edema, extensive inflammatory cell infiltration, crypt loss, and reduced goblet cell numbers. Relative to the DSS group, the BA intervention groups and the 5-ASA group showed relatively intact colonic mucosa, reduced submucosal congestion and edema, more regular gland arrangement, and significantly fewer inflammatory cells, with the DSS + BA-H demonstrating notably fewer inflammatory cells than the DSS + BA-M and DSS + BA-L groups (Fig 2A). The HI scores of DSS-induced mice were significantly higher compared with those of the control group. Meanwhile, we found that the HI scores of mice gradually decreased with the increase of BA dose (P < 0.01), and the scores of the DSS + BA-H group were comparable to those of the 5-ASA group (Fig 2B).

ELISA analysis showed that the expression levels of inflammatory factors TNF-α (Fig 2C), IL-1β (Fig 2D) and IL-6 (Fig 2E) in the colonic tissue of DSS group mice were significantly higher than those in the Control group. However, after BA intervention, the expression levels of these inflammatory cytokines in the colonic tissue were markedly reduced. Among them, the DSS-BA-H group exhibited the most pronounced decrease in inflammatory cytokines.

### BA modulates the gut microbiota structure in DSS-induced UC mice

**Fig 3:**
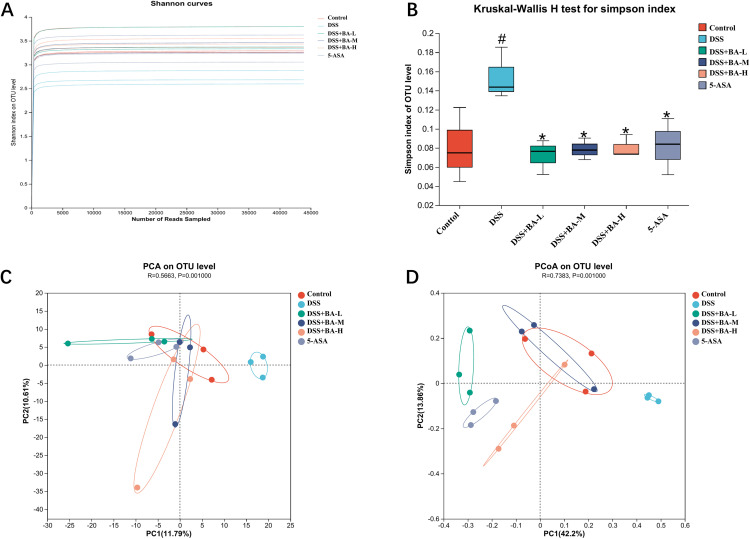
BA modulates gut microbiota diversity in DSS-induced colitis mice. **(A)** Shannon index. **(B)** Simpson index. **(C)** PCA plot. **(D)** PCoA plot. Data are presented as mean ± SEM (n = 6). #P < 0.05 vs Control; *P < 0.05 vs DSS group. Abbreviations:  DSS+BA-L (butyrate low-dose), DSS+BA-M (butyrate medium-dose), DSS+BA-H (butyrate high-dose), 5-ASA (5-aminosalicylic acid).

We examined the composition of mouse gut microbes by 16S rRNA high-throughput gene sequencing. The Shannon index rose and then leveled off indicating that our sequencing data were reasonable enough to reflect the diversity of most microorganisms in the samples (Fig 3A). Simpson index analysis revealed that the diversity of intestinal microorganisms in mice in the DSS group was significantly lower than that in the Control group, and the BA drug intervention group (Fig 3B). Beta diversity analysis showed that Control and BA drug intervention groups showed significant clustering in principal component analysis (PCA) (Fig 3C) and principal coordinate analysis (PCoA) (Fig 3D), whereas the composition of gut microorganisms of mice in the DSS group was significantly different from these two groups. This suggests that BA altered the abundance and diversity of gut flora affected by DSS.

### BA alters the composition of the gut microbiota in DSS-induced UC mice

**Fig 4:**
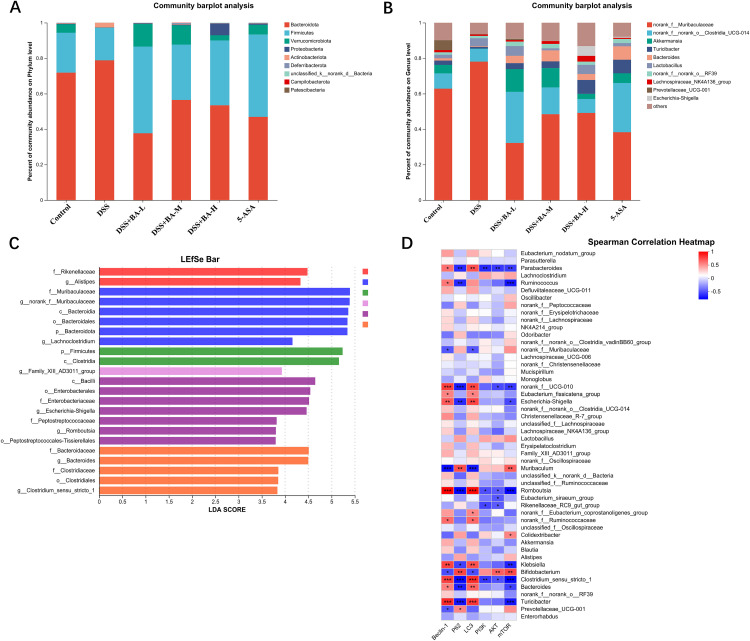
BA altered the composition of the gut microbiota in UC mice. **(A)** Analysis of species composition at the phylum level, **(B)** Analysis of species composition at the genus level, **(C)** LDA discriminant bar chart, **(D)** Correlation analysis between gut microbiota and autophagy.

We analyzed species variation in the gut microbiota at both phylum and genus levels. The results at the phylum level showed that the five most abundant phyla were *Bacteroidota*, *Firmicutes*, *Verrucomicrobiota*, *Proteobacteria*, and *Actinobacteriota*. Compared to the control group, *Bacteroidota*, *Firmicutes* were the dominant DSS group *Bacteroidota*. The relative abundance of *Firmicutes* and *Verrucomicrobiota* increased in the BA drug intervention group compared to the DSS group, while the relative abundance of *Bacteroidota* decreased significantly (Fig 4A). The results of species composition of gut microorganisms at genus level showed higher abundance of *norank_f__Muribaculaceae* and lower relative abundance of *Akkermansia* in the DSS group compared to the control group. The abundance of *Akkermansia*, *Turicibacter* and *norank__f__norank____Clostridia_UCG-014* was significantly higher in the BA drug intervention group compared to the DSS group (Fig 4B). We also performed LefSe (LDA Effect Size, LDA > 2.0) to analyze the significantly different gut microbial species in each subgroup. The results showed that *Muribaculaceae* and *Bacteroidota* were more significant in the DSS model group, and the LDA scores were > 5. In contrast to the DSS group, the significance of *Muribaculaceae* was significantly reduced in the BA drug intervention group, whereas the significance of *Peptostreptococcales* species under the phylum *Firmicutes* and *Clostridia* was increased. However, unexpectedly the significance of species under the phylum *Bacteroidota* such as *Enterobacteriaceae* and *Escherichia-Shigella* did not decrease in the BA drug intervention group (Fig 4C).

### Correlation analysis between autophagy and gut microbiota

Autophagy is a cellular activity that maintains cellular homeostasis, and in the intestinal environment, the process of autophagy removes invading microorganisms and assists the intestinal immune system. The structure and species of intestinal flora can influence autophagic activity, and changes in intestinal microbiota due to dysbiosis can stimulate or hinder autophagy. To investigate whether the effect of BA on the DSS-induced UC model is related to autophagy, we found a close relationship between the abundance of 19 bacterial genera and the expression of autophagy-associated proteins by Spearman correlation analysis between the fecal gut microbiota and autophagy-associated proteins in mice. Notably, *Parabacteroides*, *Turicibacter* and *Romboutsia* were negatively correlated with the expression of autophagy-critical proteins, whereas *Muribaculum* and *Bifidobacterium* were positively correlated with the expression of autophagy-critical proteins (Fig 4D).

### BA affects UC by blocking the PI3K/AKT/mTOR pathway and activating autophagy

**Fig 5:**
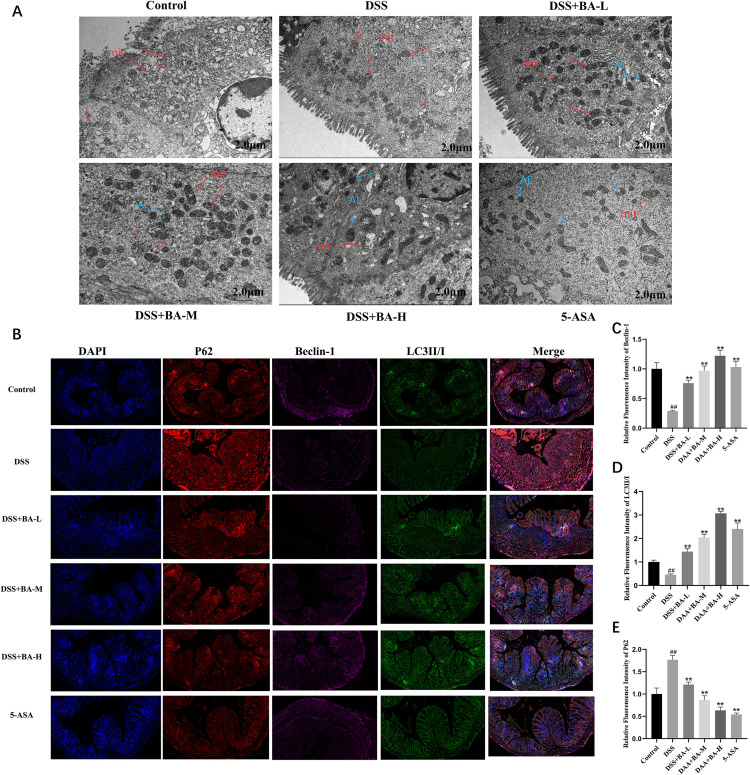
BA alleviates colitis by regulating autophagy. **(A)** TEM images of autophagic vesicles. **(B)** Immunofluorescence staining of P62, Beclin-1, and LC3II/I in colon tissue. **(C–E)** Quantification of **(C)** Beclin-1, **(D)** LC3II/I, and **(E)** P62 fluorescence intensity. Data are mean ± SEM; #P < 0.05, ##P < 0.01 vs Control; *P < 0.05, **P < 0.01 vs DSS group.

To understand whether the effect of BA on DSS-induced UC is through whether it involves the PI3K/AKT/mTOR pathway, a key pathway of autophagy, firstly, we observed the microstructure of mouse colon tissues by electron microscope. The mitochondrial membranes were intact, and mitochondrial inner cristae were clear and structurally intact in the supracolonic tissues of mice in Control group. The structure of mitochondrial inner cristae in the colonic tissues of mice in the DSS group was reduced or even disappeared, the mitochondrial swelling volume increased, and the mitochondrial cristae had a fracture condition. In the BA drug intervention group and 5-ASA group, not only mitochondrial damage of different conditions but also the formation of autophagic vesicles and autophagic lysosomes were observed in the colonic epithelial cells of mice (Fig 5A).

Then we found by immunofluorescence staining of autophagy key proteins Beclin-1, P62 and LC3II/I proteins (Fig 5B) that the expression of Beclin-1 (Fig 5C) and LC3II/I (Fig 5D) was reduced and the expression of P62 proteins (Fig 5E) was significantly higher in the colon tissues of mice in the DSS group. Compared with the DSS model group, the expression levels of Beclin-1 and LC3II/I were significantly increased and the expression level of P62 protein was significantly decreased in the BA drug intervention group (Fig 5C-E).

**Fig 6:**
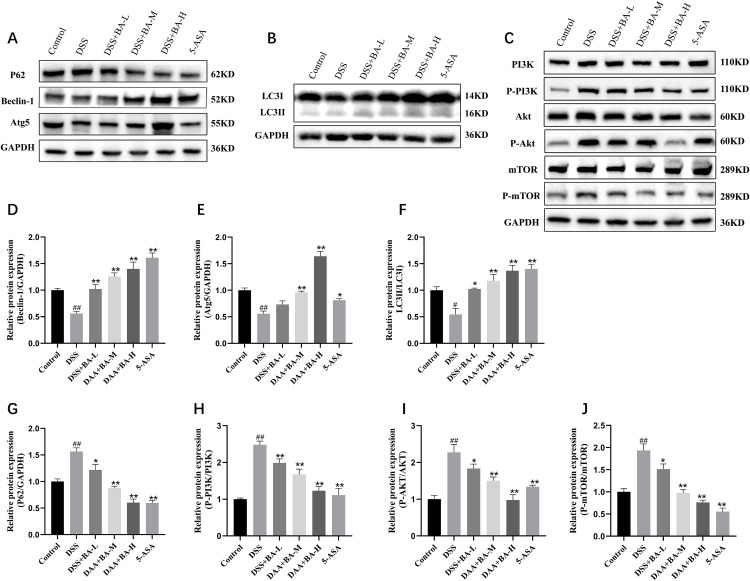
BA activates autophagy by inhibiting the PI3K/AKT/mTOR pathway in colitis. **(A) (B)** Western blot of autophagy-related proteins. **(C)** Western blot of PI3K/AKT/mTOR pathway proteins. **(D–J)** Quantitative analysis of **(D)** Beclin-1, **(E)** Atg5, **(F)** LC3II/I, **(G)** P62, **(H)** p-PI3K/PI3K, **(I)** p-AKT/AKT, and **(J)** p-mTOR/mTOR protein levels. Data are mean ± SEM; #P < 0.05, ##P < 0.01 vs Control; *P < 0.05, **P < 0.01 vs DSS group.

Finally, the expression levels of autophagy key proteins Beclin-1, Atg5, P62 and LC3II/I proteins (Fig 6A and B) and autophagy negative regulatory pathway-related PI3K, AKT and mTOR proteins in mouse colon tissues were further detected by Western blot (Fig 6C). The results revealed that the expression levels of Beclin-1 (Fig 6D) and Atg5 proteins (Fig 6E) and the ratio of LC3II/I (Fig 6F) were significantly lower, the expression of P62 (Fig 6G) was significantly higher, and the expression ratios of the pathway proteins P-PI3K/PI3K (Fig 6H), P-AKT/AKT (Fig 6I), and P-mTOR/mTOR (Fig 6J) were significantly increased in the colonic tissues of mice in the DSS group. Compared with the DSS model group, the BA drug intervention group up-regulated the expression of autophagy-related proteins Beclin-1 and Atg5 and the ratio of LC3II/I (Fig 6D-F), and suppressed the expression of P62 proteins and pathway proteins P-PI3K/PI3K, P-AKT/AKT and P-mTOR/mTOR (Fig 6G-J). The trend of DSS-BA-H histone changes was more obvious. This suggests that BA can activate autophagy shipment is occurring by inhibiting the PI3K/AKT/mTOR signaling pathway.

## Discussion

Short-chain fatty acids (SCFAs) are generated as end-products of anaerobic fermentation of dietary fibers by gut microbiota, with primary components including acetate, propionate, and BA [22]. Among these, BA has attracted substantial attention owing to its nutritional effects on intestinal epithelial cells and anti-inflammatory properties. Existing research confirms that oral supplementation with BA can effectively reduce the DAI in patients with UC, decrease endoscopic and histological scores, and promote mucosal healing [23], but the underlying mechanism of action is unclear. Therefore, we established a mouse model of UC by DSS and intervened with BA medication to investigate the mechanism of action of BA on UC. For the first time, we explored the mechanism of action of BA on UC in terms of gut microbiota and autophagy. The results showed that BA may alleviate UC by regulating the intestinal flora and inducing autophagy in intestinal epithelial cells.

Intestinal inflammation is the most prominent feature of ulcerative colitis, and BA, as a short-chain fatty acid, has been most studied for its anti-inflammatory effects. In our study, by administering BA to UC mice, we found that the intervention of BA significantly improved intestinal inflammation, repaired mucosal damage, and lowered DAI in UC mice. We divided BA into three dose groups, low, medium and high, and observed that with the increase of BA dose, the intestinal damage in UC mice was reduced and the inflammatory response was alleviated, in which the efficacy of DSS + BA-H group (1200 mg/kg) was similar to that of 5-ASA group, which was consistent with the findings of Yan et al [24]. Meanwhile, BA could also alleviate the intestinal inflammatory response in UC mice by reducing the production of inflammatory factors TNF-α, IL-1β and IL-6. Above These results suggest that BA has potential therapeutic effects on UC.

Intestinal flora is an important factor in maintaining the homeostasis of the colonic epithelial microenvironment. It is well documented that disturbances in the intestinal flora accelerate UC disease progression. BA is also closely related to the gut microbiota, with BA supplementation modulating the composition of the intestinal microbiota, which in turn produces BA through the fermentation of dietary fibers. These interactions contribute to the maintenance of the integrity of the intestinal barrier [25]. To investigate the effect of BA on the intestinal flora of UC, we found that the relative abundance of butyric acid flora was significantly reduced in DSS-treated mice as detected by 16S rRNA technique. In contrast, BA treatment effectively increased the abundance and diversity of protective bacterial communities in UC mice. In addition, the abundance of the anti-inflammatory bacterium *Akkermansia*, and the butyric acid-producing probiotic *Turicibacter* in the intestinal tract of mice was increased after BA intervention [26], whereas the abundance of the bacteria *Norank_f__Muribaculaceae*, and *Bacteroidota*, which have been associated with the promotion of inflammation and potentially damaging the intestinal mucosal barrier, was decreased [27]. Our findings in mice are consistent with human studies that report a depletion of butyrate-producing bacteria, such as *Faecalibacterium prausnitzii*, in UC patients [28]. The abundance of *Akkermansia* is often Positive correlated with disease severity in human IBD [29], the increase of *Akkermansia* observed in our BA-treated mice was particularly notable. This parallel suggests that the ability of BA to restore a beneficial microbial community in mice may mirror its potential to correct similar dysbiosis in human UC, which could be a key component of its therapeutic effect.

Recent studies demonstrate that autophagy dysfunction may lead to dysbiosis of the gut microbiota, subsequently triggering subsequent intestinal epithelial dysfunction and inflammatory cascades [30]. Autophagy in intestinal epithelial cells facilitates the clearance of invasive gut bacteria and restoration of microbial homeostasis [31]. In the present study, autophagosomes and autolysosomes in the colon epithelial cells of the BA intervention group were observed by electron microscopy. Furthermore, the expression of autophagy-related proteins was upregulated in the intestinal tissues of mice in the BA group, suggesting that BA promotes autophagy in intestinal epithelial cells. Importantly, the close relationship between changes in intestinal flora and autophagy was verified by Spearman correlation analysis. The PI3K/AKT/mTOR pathway, as a classic autophagy regulatory pathway, has been shown to be involved in butyrate-induced autophagy in intestinal epithelial cells [32]. Prior studies indicate that inhibiting this pathway can activate autophagy in intestinal epithelial cells to alleviate colitis caused by Clostridium difficile infection [33]. In this study, we observed downregulated expression of PI3K/AKT/mTOR pathway-related proteins in the BA group. Spearman correlation analysis confirmed the close relationship between changes in intestinal flora and pathway protein expression. Therefore, BA-mediated gut microbiota modulation is likely associated with inhibition of the PI3K/AKT/mTOR pathway, which induces autophagy in intestinal epithelial cells.

However, this study has limitations. First, the study did not utilize mTOR pathway agonists or inhibitors to validate the role of the PI3K/AKT/mTOR pathway in UC pathogenesis. Second, the safety profile and potential toxicity of BA in UC mice was not assessed. Finally, the relationship between gut microbiota, autophagy, and the PI3K/AKT/mTOR pathway was not explored in UC patients.

In summary, the present study demonstrates that BA perhaps confers protection against UC in mice by promoting intestinal epithelial cell autophagy and restructuring the gut microbiome via inhibition of the PI3K/AKT/mTOR pathway.

## Supporting information

S1 File Minimal data set. This dataset contains all the variables needed to reproduce the results of this research report, as well as all the raw measurement data from the experimental group.
(PDF)

S2 File Original images for blots. This file contains the original, uncropped, and unprocessed blot images for all Western blot analyses. Each image is labeled with the target protein (e.g., Akt, p-Akt, GAPDH). Molecular weight markers are visible in all images.
(PDF)
