# Supplementary material for: Butyrate ameliorates DSS-induced ulcerative colitis in mice by facilitating autophagy in intestinal epithelial cells and modulating the gut microbiota through blocking the PI3K-AKT-mTOR pathway
Source: PLoS One. 2025 Dec 11;20(12):e0337214. doi: 10.1371/journal.pone.0337214 (PMC12697976; doi:10.1371/journal.pone.0337214)
Supplement: S1 File — This dataset contains all the variables needed to reproduce the results of this research report, as well as all the raw measurement data from the experimental group. (PDF) [file pone.0337214.s001.pdf]

Figure 1B Body weight changes（g） minimal data set:

Days	Control				
0	100	100	100	100	100
1	101	100	101	104	102
2	101	100	100	104	103
3	102	101	102	105	104
4	103	101	103	106	106
5	101	102	100	103	103
6	100	99	98	104	103
7	104	104	103	105	105
8	102	102	102	104	104
9	104	106	104	107	106

Days	DSS				
0	100	100	100	100	100
1	99	98	103	105	101
2	100	90	101	104	98
3	95	96	95	96	90
4	94	85	94	90	89
5	82	82	88	88	84
6	83	80	84	84	80
7	80	80	81	76	78
8	78	73	76	77	75
9	74	70	74	72	76

Days	DSS+BA-L				
0	100	100	100	100	100
1	103	99	104	101	98
2	104	97	102	103	98
3	98	93	97	93	89
4	94	83	96	95	91
5	90	89	90	88	80
6	90	87	84	88	72
7	86	82	82	86	75
8	84	81	80	84	74
9	83	78	79	83	74

Days	DSS+BA-M				
0	100	100	100	100	100
1	100	100	102	102	100
2	101	103	102	100	102
3	95	97	94	90	95
4	90	90	100	89	89
5	90	89	90	89	90
6	87	89	84	84	86
7	86	89	80	81	83
8	84	86	79	83	84
9	82	83	78	82	83

Days	DSS+BA-H				
0	100	100	100	100	100
1	99	99	105	102	102
2	102	88	102	93	103
3	92	95	102	99	95
4	95	91	97	91	94
5	92	89	92	91	92
6	94	87	92	93	93
7	92	85	90	92	91
8	91	83	91	89	90
9	89	84	91	90	91

Days	5-ASA				
0	100	100	100	100	100
1	101	100	102	98	96
2	101	97	102	98	93
3	91	93	97	94	94
4	98	87	95	86	91
5	91	91	94	89	89
6	90	91	93	87	86
7	89	86	90	82	87
8	88	87	88	80	86
9	89	87	87	78	88

Figure 1C Colon length（cm） minimal data set:

Control	DSS	DSS+BA-L	DSS+BA-M	DSS+BA-H	5-ASA
9.8	4	7.2	7.7	9.8	9.2
10	5.2	6.8	8.1	9.4	8.2
9.9	6.3	6.9	7	9.2	8.7
9.5	6.5	6.4	7.6	9.5	8.6
8.9	6	7	8.5	8.3	8.6
9.7	5.6	6.9	7.2	7.8	8
9.633	5.600	6.867	7.683	9.000	8.550

The values in blue font are the averages.

Figure 1D Disease activity index (DAI) minimal data set:

Days	Control				
0	0	0	0	0	0
1	0	0	0	0	0
2	0	0	1	0	0
3	0	0	0	0	0
4	0	0	0	0	0
5	1	0	1	1	1
6	1	1	1	0	0
7	0	0	0	0	0
8	1	1	1	1	1
9	0	0	0	0	0

Days	DSS				
0	0	0	0	0	0
1	1	1	0	0	0
2	1	2	2	1	2
3	4	2	3	3	3
4	5	8	3	7	4
5	9	7	6	9	6
6	7	9	9	9	7
7	9	8	9	10	8
8	7	8	9	5	9
9	7	6	7	8	6

Days	DSS+BA-L				
0	0	0	0	0	0
1	0	1	0	0	1
2	3	2	3	2	2
3	2	3	3	5	5
4	4	6	2	3	3
5	8	6	5	10	5
6	8	8	10	7	10
7	9	2	8	8	7
8	5	5	6	5	8
9	4	4	5	4	5

Days	DSS+BA-M				
0	0	0	0	0	0
1	0	0	0	1	1
2	0	1	0	1	3
3	4	2	2	2	2
4	2	4	3	3	5
5	7	4	6	4	4
6	9	7	6	10	9
7	7	6	6	8	7
8	5	4	5	6	5
9	4	4	3	4	3

Days	DSS+BA-H				
0	0	0	0	0	0
1	1	1	0	0	0
2	4	2	3	5	3
3	5	2	2	4	5
4	7	5	3	5	6
5	8	9	7	3	8
6	7	8	9	6	8
7	5	6	5	5	6
8	4	4	2	4	3
9	3	2	1	2	2

Days	5-ASA				
0	0	0	0	0	0
1	0	0	0	0	0
2	0	0	0	1	1
3	4	1	3	4	5
4	7	7	4	6	4
5	4	4	8	6	5
6	9	8	10	9	9
7	5	6	7	7	6
8	3	4	4	7	4
9	2	4	2	5	3

Figure 2B Histopathological score minimal data set:

Control	DSS	DSS+BA-L	DSS+BA-M	DSS+BA-H	5-ASA
0	3	2	3	2	1
1	4	2	2	2	2
0	3	2	2	1	1
0	3	2	1	2	1
1	4	3	2	1	1
0	3	2	1	1	2
0.3333	3.333	2.167	1.833	1.500	1.333

The values in blue font are the averages.

Figure 2C ELISA of TNF-α minimal data set:

Control	DSS	DSS+BA-L	DSS+BA-M	DSS+BA-H	5-ASA
117.209	425.952	300.378	219.179	162.5293	115.321
178.58	395.738	294.713	292.824	144.5901	161.585
144.5901	498.6526	308.8751	230.5093	205.0168	173.8593

Figure 2D ELISA of IL-1β (D) minimal data set:

Control	DSS	DSS+BA-L	DSS+BA-M	DSS+BA-H	5-ASA
116.1222	372.518	310.3323	283.545	206.052	190.745
77.8542	431.833	323.7261	314.159	203.1819	240.493
152.4768	429.9198	273.9777	172.5675	165.8706	243.3633

Figure 2E ELISA of IL-6 minimal data set:

Control	DSS	DSS+BA-L	DSS+BA-M	DSS+BA-H	5-ASA
197.4417	396.435	283.5447	249.104	208.9221	192.658
192.6582	362.951	273.021	218.489	163.0005	208.922
200.3118	357.2106	322.7694	238.5798	162.0438	152.4768

Figure 5C Quantification of Beclin-1 minimal data set:

Control	DSS	DSS+BA-L	DAA+BA-M	DAA+BA-H	5-ASA
1.191613	0.313061	0.670021	1.10224	1.040724	0.880218
0.977731	0.283243	0.78054	0.97585	1.36338	1.002633
0.830656	0.264036	0.826036	0.831784	1.258045	1.20772

Figure 5D Quantification of LC3II/I minimal data set:

Control	DSS	DSS+BA-L	DAA+BA-M	DAA+BA-H	5-ASA
1.079091	0.43359	1.549435	1.746971	3.090529	2.139534
0.996632	0.498079	1.480335	2.224842	3.136664	2.422785
0.924277	0.431894	1.288562	2.138924	2.979855	2.654159

Figure 5D Quantification of P62 minimal data set:

Control	DSS	DSS+BA-L	DAA+BA-M	DAA+BA-H	5-ASA
1.149923	1.697359	1.263001	0.968082	0.708209	0.57135
0.891049	1.879033	1.220116	0.780916	0.558337	0.545306
0.959029	1.72109	1.153417	0.850976	0.629612	0.506365

Figure 6D Quantitative analysis of Beclin-1 protein levels minimal data set:

Control	DSS	DSS+BA-L	DAA+BA-M	DAA+BA-H	5-ASA
1.0121	0.606539	1.093084	1.307535	1.46297	1.672109
1.028877	0.55051	1.120034	1.281833	1.586615	1.653339
0.959023	0.522727	0.855637	1.169182	1.151125	1.507417

Figure 6E Quantitative analysis of Atg5 protein levels minimal data set:

Control	DSS	DSS+BA-L	DAA+BA-M	DAA+BA-H	5-ASA
0.956942	0.576518	0.686511	0.98546	1.49543	0.802823
1.001979	0.592747	0.863419	0.952686	1.622062	0.843279
1.041079	0.496462	0.643248	0.935188	1.806424	0.787373

Figure 6F Quantitative analysis of LC3II/I protein levels minimal data set:

Control	DSS	DSS+BA-L	DAA+BA-M	DAA+BA-H	5-ASA
1.098592	0.76168	1.05117	1.29647	1.541815	1.483134
1.024213	0.483995	1.02979	1.294127	1.374595	1.482809
0.877195	0.389274	0.989912	0.942408	1.187925	1.237735

Figure 6G Quantitative analysis of P62 protein levels minimal data set:

Control	DSS	DSS+BA-L	DAA+BA-M	DAA+BA-H	5-ASA
0.949867	1.637596	1.255767	0.856178	0.649682	0.547103
1.099968	1.629146	1.375645	0.909528	0.631805	0.633561
0.950165	1.423648	1.020148	0.868166	0.525207	0.608317

Figure 6G Quantitative analysis of p-PI3K/PI3K protein levels minimal data set:

Control	DSS	DSS+BA-L	DAA+BA-M	DAA+BA-H	5-ASA
0.965358	2.368976	1.90003	1.751626	1.098007	1.000184
1.028843	2.555989	2.10651	1.51541	1.30477	1.010982
1.005799	2.518393	1.962171	1.764666	1.282454	1.321899

Figure 6H Quantitative analysis of p-AKT/AKT protein levels minimal data set:

Control	DSS	DSS+BA-L	DAA+BA-M	DAA+BA-H	5-ASA
1.065	2.426236	1.921373	1.535619	1.073132	1.337796
1.053111	2.366916	1.886013	1.582215	1.046361	1.381511
0.881889	2.027117	1.696351	1.385815	0.805691	1.294952

Figure 6H Quantitative analysis of p-mTOR/mTOR protein levels minimal data set:

Control	DSS	DSS+BA-L	DAA+BA-M	DAA+BA-H	5-ASA
1.015817	2.224786	1.480586	1.036593	0.813369	0.647187
0.922552	1.829075	1.331916	0.887305	0.707119	0.50855
1.061631	1.752223	1.728917	1.003831	0.762246	0.502115
