# Supplementary figures and images for: Butyrate ameliorates DSS-induced ulcerative colitis in mice by facilitating autophagy in intestinal epithelial cells and modulating the gut microbiota through blocking the PI3K-AKT-mTOR pathway
Source: PLoS One. 2025 Dec 11;20(12):e0337214. doi: 10.1371/journal.pone.0337214 (PMC12697976; doi:10.1371/journal.pone.0337214)

Original Images for Blots/Gels: Figure 6 Original western blot:

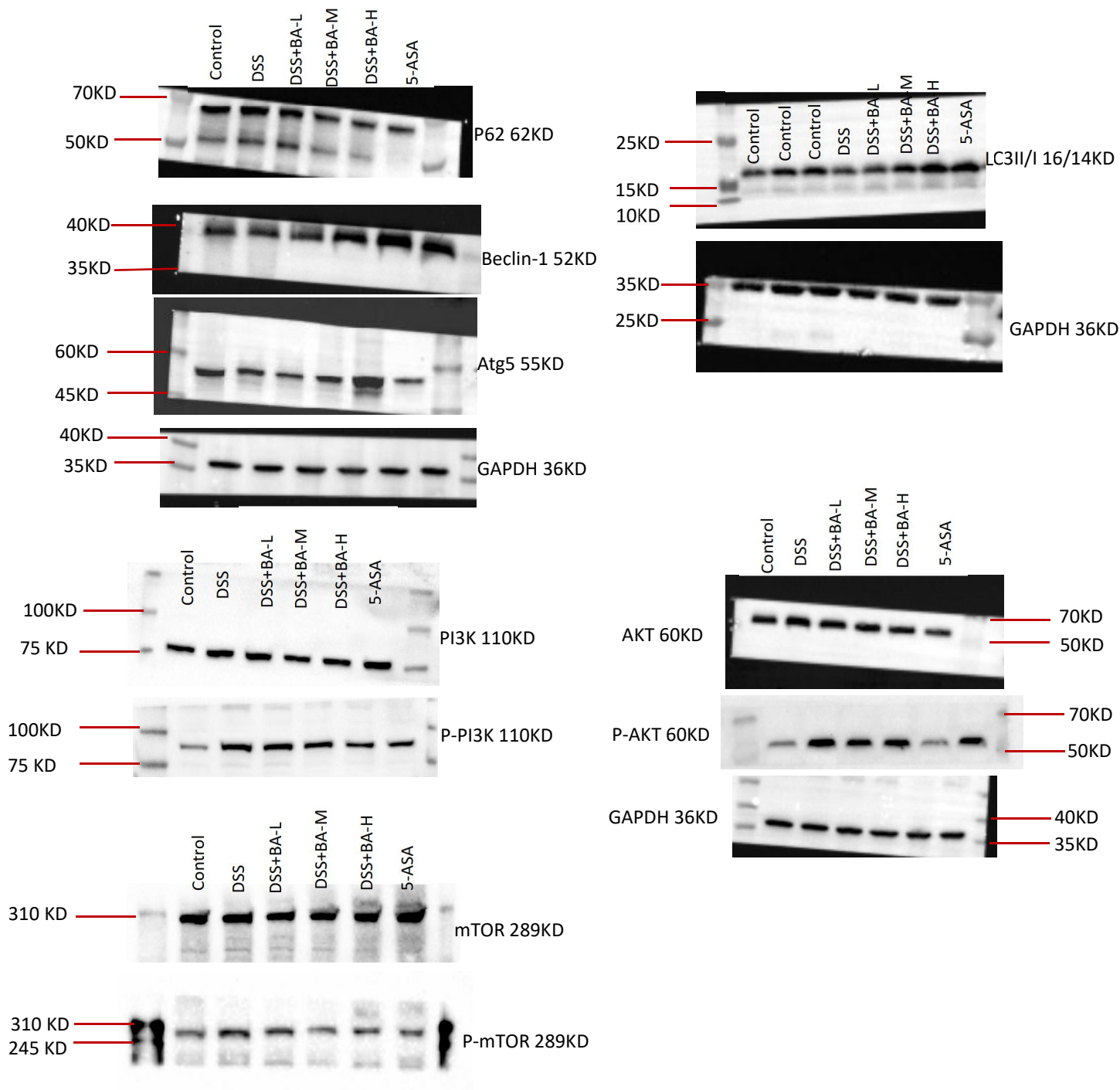

Supplement: S2 File — This file contains the original, uncropped, and unprocessed blot images for all Western blot analyses. Each image is labeled with the target protein (e.g., Akt, p-Akt, GAPDH). Molecular weight markers are visible in all images. (PDF) [file pone.0337214.s002.pdf]
